# Generation of the Antioxidant Hydroxytyrosol from Tyrosol Present in Beer and Red Wine in a Randomized Clinical Trial

**DOI:** 10.3390/nu11092241

**Published:** 2019-09-18

**Authors:** Natalia Soldevila-Domenech, Anna Boronat, Julian Mateus, Patricia Diaz-Pellicer, Iris Matilla, Marta Pérez-Otero, Ana Aldea-Perona, Rafael de la Torre

**Affiliations:** 1 Integrative Pharmacology and Systems Neurosciences Research Group, Neurosciences Research Program, Hospital del Mar Medical Research Institute (IMIM), Department of Experimental and Health Sciences, University Pompeu Fabra, 08003 Barcelona, Spain; natalia.soldevila01@gmail.com (N.S.-D.); aboronat@imim.es (A.B.); 2 Integrative Pharmacology and Systems Neurosciences Research Group, Neurosciences Research Program, Hospital del Mar Medical Research Institute (IMIM), School of Medicine, Universitat Autònoma de Barcelona, 08193 Bellaterra, Spain; jmateus@imim.es (J.M.); 62234@parcdesalutmar.cat (P.D.-P.); 3 Integrative Pharmacology and Systems Neurosciences Research Group, Neurosciences Research Program, Hospital del Mar Medical Research Institute (IMIM), 08003 Barcelona, Spain; imatilla@imim.es (I.M.); mperez2@imim.es (M.P.-O.); aaldea@imim.es (A.A.-P.); 4 Integrative Pharmacology and Systems Neurosciences Research Group, Neurosciences Research Program, Hospital del Mar Medical Research Institute (IMIM), Department of Experimental and Health Sciences, University Pompeu Fabra, CIBER de Fisiopatología de la Obesidad y la Nutrición (CIBEROBN), Instituto de Salud Carlos III, 28029 Madrid, Spain

**Keywords:** beer, wine, tyrosol, hydroxytyrosol, alcohol, metabolism, *CYP2A6*, *CYP2D6*, polygenic activity score

## Abstract

Beer and wine contains the simple phenol tyrosol (TYR) which is endogenously converted into hydroxytyrosol (HT), one of the strongest dietary antioxidants, by *CYP2A6* and *CYP2D6* polymorphic enzymes. We investigated in humans the rate of this bioconversion after beer and red wine (RW) intake. In a single blind, randomized, crossover, controlled clinical trial (*n* = 20 healthy subjects), we evaluated TYR absorption and biotransformation into HT following a single dose of (i) RW, (ii) Indian pale ale beer (IPA), (iii) blonde beer, and (iv) non-alcoholic beer (free). Individuals were genotyped for *CYP2A6* and *CYP2D6*, and a polygenic activity score (PAS) was derived. RW triggered the highest increase in total TYR recovered, followed by IPA, blonde, and free beers. Although the HT content in beer was minimal, an increase in HT production was observed in all beers following TYR in a dose-response manner, confirming TYR to HT biotransformation. Sex differences were identified in the rate of the conversion following RW. PAS scores correlated linearly with the recoveries of HT (HT:TYR ratios) after RW intake. In conclusion, after beer and RW consumption, TYR is absorbed and endogenously biotransformed into HT. This mechanism could be modulated by sex, genetics, and matrix components.

## 1. Introduction

The simple phenol hydroxytyrosol (HT) is the most abundant phenolic compound in extra virgin olive oil (EVOO) [1] and thus one of the strongest antioxidants present in the Mediterranean diet [2]. In 2006, the EUROLIVE clinical trial described a dose-dependent relationship between the EVOO phenolic fraction and beneficial health effects, particularly regarding protection against low density lipoprotein (LDL) oxidation [3]. In 2011, the European Food Safety Authority (EFSA) released a health claim concerning the benefits of the daily ingestion of 5 mg of HT and its derivates in olive oil in the prevention of LDL oxidation [4].

Fermented beverages such as wine and beer are sources of the simple phenol tyrosol (TYR) [5]. TYR is produced during fermentation as a byproduct of tyramine metabolism and can be endogenously converted into HT in the human body after ingestion [6]. This biotransformation is of relevance since in vitro studies have demonstrated that HT possesses higher antioxidant capacity than TYR [7]. Both molecules share the same structure; however, TYR lacks a catechol group (-OH). The extra catechol group of HT enhances its capacity to stabilize free radicals. In the case of TYR, the single hydroxyl group does not provide any direct antioxidant activity [8].

TYR hydroxylation to produce HT is catalyzed by two isoforms of cytochrome P450 (CYP): *CYP2A6* and *CYP2D6* [9]. Their enzymatic activities present considerable inter-individual variation in the general population, primarily due to genetic polymorphisms [10]. To date, few investigations have examined the impact of *CYP2A6* and *CYPD26* genotypic profiles on the metabolism of dietary phenolic compounds [11].

TYR bioavailability and HT biotransformation have never been described within the context of beer consumption. Better knowledge of their metabolism would help to determine beer contribution to human antioxidant status. Therefore, we designed a study aimed at (i) confirming TYR absorption and biotransformation into HT within the context of beer consumption, (ii) determining the effect of alcohol present in beer on the absorption of TYR, and (iii) analyzing the impact of sex and *CYP2A6/CYP2D6* polymorphisms on TYR conversion efficiency into HT.

## 2. Materials and Methods

### 2.1. Wine and Beer Characteristics

The red wine (RW) used in the present study was Jardins Negre 2017 (14% Alc. Vol) from the Castillo de Perelada S.A. winery (Girona, Spain). Blonde ale beer (Blonde; 4.5% Alc. Vol), and India Pale Ale (IPA; 8.5% Alc. Vol) were provided by Cervesa Espiga (Sant Llorenç d’Hortons, Spain). The non-alcoholic beer (Free) was Aigua de Moritz (0.0% Alc. Vol) manufactured by Cervezas Moritz S.A (Barcelona, Spain). All four products can be obtained from the Spanish market. TYR and HT content of administered treatments was determined using LC/MS-MS and is detailed in Table 1.

Beer choice was based on alcohol and TYR content in comparison to RW. IPA belongs to a group of beers with a high content of TYR and its dose was matched to RW in terms of alcohol. Both treatments resulted in the same amount of alcohol and both were high in TYR. Blonde beer was selected as a standard beer for its content in TYR and alcohol (about half of the previous two conditions). Finally, free beer was chosen, matching its TYR content to blonde beer, to assess the contribution of alcohol to TYR absorption. The quantities of RW and beers administered were based on normally consumed amounts in the Spanish population and followed moderate alcohol consumption recommendations.

### 2.2. Participants

A total of twenty healthy subjects (50% women) were recruited by word of mouth from June 2018 to February 2019 by the Clinical Research Unit (CRU) of the Hospital del Mar Medical Research Institute (IMIM, Barcelona, Spain). Eligibility criteria were: Healthy individuals aged from 18 to 45 years with a recreational consumption of alcohol. Exclusion criteria were: Severe chronic diseases, multiple allergies, intestinal, hepatic or renal conditions that may affect normal phenol metabolism; restrictive diets and antioxidant supplements; a previous history of alcohol hypersensitivity/intolerance; consumption of >50 g of alcohol per day; body mass index (BMI) <18.5 or >30.0 kg/m^2^; pregnancy/breastfeeding; smoking habit; and illiteracy.

Written informed consent was obtained from all screened participants prior to any clinical procedure. Subjects underwent a general physical examination, routine laboratory test, urinalysis, and a 12-lead electrocardiogram presenting results within normal values. The trial was performed following good clinical practices and in accordance with the Helsinki declaration. It was approved by our local ethical committee (CEIm-Parc de Salut Mar) and registered in ClinicalTrials.gov: NCT03614520.

### 2.3. Study Design

The study was a single blind, randomized, crossover, controlled clinical trial. Participants were randomly assigned to one order of administration to receive a single dose of: RW (150 + 100 mL of water), IPA (250 mL), blonde (250 mL), and free beer (250 mL). Treatments were administered on four separate experimental sessions of 6 h duration. On the session day, participants arrived in fasting conditions to the CRU. Baseline urinary samples were obtained the two first hours (-2–0 h). Participants were administered a single dose of the treatment in opaque glasses. Urine was collected post-administration at the following time fractions: 0–2 h, 2–4 h, 4–6 h, 6–12 h, and 12–24 h. The subjects were given a light meal (cheese sandwich) after 2h post-administration, and remained at the CRU until 4 h post-administration. Session days were preceded by a 72 h wash-out period, in which participants followed a diet low in phenols and abstained from any alcoholic drink (see supporting information). Total urinary volume and pH were measured in all urine samples. A blood sample was collected at the end of the study for DNA sampling; tubes were centrifuged (1700 g, 15 min, 4 °C) to isolate the buffy coat.

### 2.4. Volunteer Genotyping

*CYP2A6* and *CYP2D6* genotypes were determined for the eighteen volunteers who consented to DNA sample extraction. Genomic DNA was isolated from the buffy coat using the QIAamp DNA blood mini kit (Qiagen, Dusseldorf, Germany).

Volunteers were genotyped for the most common allelic variants in the Spanish population of *CYP2A6* (* 2, * 4, * 9, * 12, * 1 xN (xN indicates more than one copy) and *CYP2D6* (* 2, * 3, * 4, * 5, * 9, * 10, * 34 * 35, * 41, 1 xN, 2 xN, 35 xN) [12,13] using the TaqMan allelic discrimination method (Applied Biosystems, Foster City, CA, USA). *1 allele was assumed when none of the tested allelic variants were detected. The tested allelic variants were categorized according to functionality into: non-function, reduced-function, functional, and increased-function. A scoring system was established for each enzyme based on the method described by Gaedigk et al. [14] for *CYP2D6* and extrapolated to CYP2A6. Tested allelic variants were categorized according to functionality into non-functional, reduced-function, normal function, and increased function (Table 2). A score of 0, 0.5, or 1 was assigned for the presence of each allele, obtaining a final activity score ranging from 0 to 2 for each gene. The allelic score could later be multiplied in the case of duplications. Lastly, both activity scores were added to obtain a final PAS for each individual as previously described [11].

### 2.5. TYR and HT Urinary Recovery

Urinary recovery of TYR and HT metabolites was quantified by means of a solid-phase extraction followed by LC-MS/MS analysis as previously described [15]. The methodology identified and quantified free forms of TYR, HT, homovanillyl alcohol (Hval), and HT-acetate; TYR-4-sulphate, TYR-4-glucuronide, HT-3-sulphate, HT-4-sulphate, HT-acetate-3-sulphate, HT-acetate, HT-3-glucuronide, HT-4-glucuronide, and Hval-4-glucuronide. Briefly, 0.5 mL of urine was stabilized with 1 mL of phosphoric acid (4% *v/v*) and spiked with 10 µL of the internal standard mix (containing 10 µg/mL of 3-(4-hydroxyphenyl)-1-propanol, HT-d3, 3-(4-hydroxyphenyl)-1-propanol glucuronide, and HT-1′-O-sulphate). Thereafter, samples were subjected to a solid-phase extraction using 3mL Oasis HLB columns and 60 mg cartridges from Waters Corporation (Milford, MA, USA). Samples were loaded into cartridges, washed with 2 mL of purified water, and the compounds of interest eluted with 2mL of methanol. Methanol extracts were dried under nitrogen stream (<15 psi, 29 °C). Dry extracts were reconstituted with a mixture containing 91% ammonium acetate (0.01%, pH 5) and 9% pure methanol.

Urinary concentrations were extrapolated by means of a calibration curve. Calibrators and blank samples were prepared using blank urine spiked with the compounds of interest. Finally, to calculate compound recovery, urinary concentrations were standardized by the total volume of urine collected in each time fraction.

TYR and HT metabolites were identified and quantified with an Agilent 1200 series HPLC system coupled to a triple quadrupole (6410 Triple Quad LC/MS) mass spectrometer with an electrospray interface from Agilent Technologies (Santa Clara, CA, USA). Mobile phases were (1) water with 0.01% (*v/v*) ammonium acetate (pH 5), and (2) pure methanol. An Acquity UPLC^®^BEH C18 column (100 × 3 mm i.d., 1.7 µm particle size) from Waters Corporation (Milford, MA USA) was used at 40 °C for the chromatographic separation.

### 2.6. Ethyl Glucuronide (EtG) Determination

Urinary EtG excretion, an alcohol consumption biomarker, was quantified using a dilute-and-shoot approach [16]. Briefly, 30 µL of urine was spiked with 10 µL of internal standard containing 10 g/mL ethyl-glucuronide-d5 and diluted into a final volume of 150 µL with mobile phase. Identification and quantification were performed with an LC/MS-MS Agilent 1200 series HPLC (Agilent technologies, Santa Clara, CA USA) coupled to a triple quadrupole with an electrospray (6410 Triple Quad LC/MS). Mobile phase compositions were: (1) water with 0.1% (*v/v*) formic acid, and (2) acetonitrile with 0.1% (*v/v*) formic acid. To perform the chromatographic separation an Acquity UPLC^®^ BEH C18 column with a 1.7 µm particle size, 3 × 100 mm (Waters, Milford, MA, USA) was employed.

### 2.7. Sample Size and Power Analysis

A total sample size of 20 participants (10 of each sex) would allow 85% power to detect a statistically significant difference in total HT recovery of 1.2 µmoL, assuming an adjusted type I error of 0.05, two-sided. Based on our previous experiments, the mean (SD) of HT and its metabolites excreted within 24 h post-administration of 270 mL of white wine is 5.6 (5.4) µmoL, with a difference of 1.2 µmoL compared to placebo. Thus, we assumed a standard deviation of total HT recovery of 5.4 µmoL. The sample size was calculated with the GLIMMPSE software [17] considering the multiple comparisons of the study.

### 2.8. Statistical Analysis

Analyses were performed with R, version 3.0.2 [18]. R packages used were “multcomp”, “nlme”, and “ggplot2”. Linear mixed effect models were devised to perform comparisons among treatments. Tukey’s HSD (honestly significant difference) test was used to carry out post-hoc pairwise comparisons. Pearson correlation coefficient was calculated to evaluate the existence of linear associations between the dose of TYR administered and total TYR and HT recoveries. Significance was set as *p* < 0.05.

## 3. Results

### 3.1. Characteristics of Study Participants

A total of 20 individuals successfully finished the study. Volunteer’s characteristics are detailed in Table 3. Men volunteers presented higher weight and BMI than women volunteers (*p* < 0.050).

Eighteen volunteers (two declined genetic testing) were genotyped for *CYP2A6* and *CYP2D6*; from this information, a PAS was calculated, ranging from 2 to 4. Sex was not equally distributed among PAS groups: Men presented higher values of PAS than women (*p* < 0.001).

Volunteers’ compliance with the low phenolic diet and alcohol abstinence was assessed in baseline urines. Baseline EtG levels revealed that one male volunteer did not follow the alcohol abstinence requested in the wash-out periods in two of the four study visits corresponding to blonde and free beers (baseline EtG levels were 2.8 and 12.9 µmoL, respectively). Consequently, these two visits from this volunteer were removed from the analysis.

### 3.2. TYR and HT Metabolite Recovery in Urine

#### 3.2.1. Total TYR and HT

Urinary recoveries of total TYR and total HT in different urinary fractions, and the accumulated TYR and HT after 6 and 24 h following each treatment, are presented in Table 4. After 6 h post-administration, RW triggered the highest increase in total TYR with a mean (SD) of 6.2 (2.9) µmoL. TYR recovery following IPA and blonde beer was similar, with a mean (SD) of 3.1 (2.4) and 3.3 (1.8) µmoL, respectively. Free beer presented the lowest recovery of TYR: 0.3 (1.0) µmoL (*p* < 0.001 compared to the other treatments). In terms of total HT excretion, within the first 6 h the highest recoveries were observed following RW, with a mean (SD) of 3.1 (1.3) µmoL (*p* < 0.001 vs. all treatments). The highest HT recovery following beer intake was obtained after IPA (1.0 µmoL, SD 0.6), followed by blonde, and finally free beer. After 24 h post-administration, the same trend was observed regarding total TYR metabolites, RW was the treatment with the highest recovery and free beer the lowest (*p* < 0.001). In terms of total HT recovery at 24 h, the highest recoveries were also observed following RW. Free beer HT recovery (2.3 µmoL, SD 1.6) was higher than blonde beer (1.5 µmoL, SD 0.7) but lower than IPA (2.8, SD 1.4). Baseline total TYR and HT were low (median and interquartile rank were <0.1 µmoL) and did not differ significantly among interventions (*p* = 0.631 and *p* = 0.089, respectively).

Most TYR recovered in urine was excreted within the first 6 h post-administration: approximately 90% after RW, IPA, and blonde beers, and 30% following free beer. Regarding total HT, these values were approximately 65% for RW, 35% for IPA and blonde beers, and 16% for free beer. Treatments differed in total TYR and total HT excretion in all urinary fractions except for the fraction 12–24 h in the case of TYR, and the fractions 6–12 and 12–24 h in the case of HT.

Table 5 shows the mean percentage of the administered TYR dose recovered in urine in the form of TYR and HT following each treatment. TYR dose was recovered at 6 h post-administration in a range of 3.5% to 43%, free beer being the lowest and blonde beer the highest. The percentages of the doses recovered in the form of HT at 6 h post-administration ranged from 5.6% to 11.3%, RW presenting the highest recovery and IPA beer the lowest. Blonde beer was the treatment with highest recovery of the administered TYR (50.5%; CI95% 39.5–61.5%), in the form of both TYR and HT, followed by RW, IPA, and free beers.

**Figure 1:**
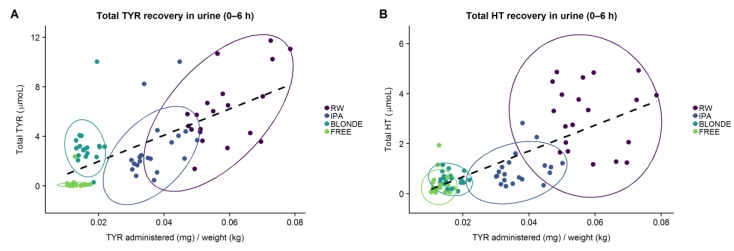
Correlation between tyrosol (TYR) administered adjusted by body weight and total TYR 6 h recovery (**A**) and total hydroxytyrosol (HT) 6 h recovery (**B**) following each treatment.

Figure 1 shows the correlation between the dose of TYR administered adjusted by body weight and the 0–6 h total TYR and HT recovery. There was a linear relationship between the dose of TYR and total TYR (r = 0.691, *p* < 0.001) and total HT (r = 0.737, *p* < 0.001) urinary recoveries.

#### 3.2.2. TYR and HT Metabolites

Differences among treatments were observed in all TYR metabolites (Supplementary Table S1): free TYR, TYR-4-sulphate, and TYR-4-glucuronide (*p* < 0.001). The most abundant form for all treatments was TYR-4-glucuronide; the contribution of the free TYR form was minor (around 6%). The observed increased HT metabolite was HT-4-sulfate (*p* < 0.001), with a minor contribution of free HT (around 8%) (*p* < 0.004) and Hval-4-glucuronide (*p* < 0.001at 6 h and *p* = 0.047 at 24 h). HT-acetate-3-sulphate, HT-acetate, HT-3-glucuronide/HT-4-glucuronide, and free Hval were not statistically different among treatments (*p* > 0.1). Supplementary Table S2 contains TYR and HT metabolite urinary recoveries by fractions of 0–2, 2–4, 4–6, 6–12, and 12–24 h.

#### 3.2.3. Sex Dimorphism in TYR and HT Metabolism

**Figure 2:**
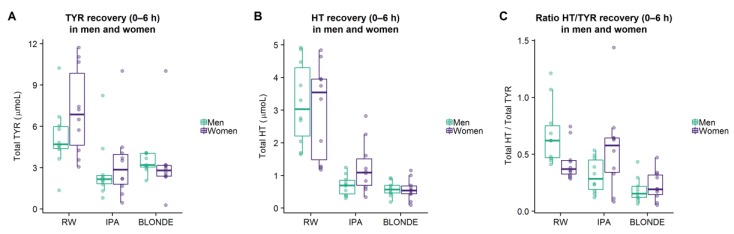
Total tyrosol (TYR) (**A**), total hydroxytyrosol (HT) (**B**), and the ratio between total HT:total TYR recoveries (**C**) collected from 0 to 6 h for different interventions stratified by sex.

Total TYR and total HT recoveries (0–6 h) in urine were studied separately in men and women. No significant results were observed with respect to total metabolites although distinct marginal trends were found following RW and IPA beer. TYR recovery was marginally greater in women compared to men (*p* = 0.099 for RW) (Figure 2A). HT recovery was also marginally higher in women compared to men following IPA beer (*p* = 0.054) (Figure 2B). A ratio between recoveries of total HT and total TYR metabolites (HT/TYR ratio) was calculated to assess the efficiency of TYR to HT conversion between men and women (Figure 2C). Statistically significant differences were observed in the case of RW within the first 6 h in which men presented greater ratios than women (*p* = 0.026) (Figure 2C). Statistical analyses were adjusted by PAS.

### 3.3. Interaction between PAS and TYR and HT Metabolism

**Figure 3:**
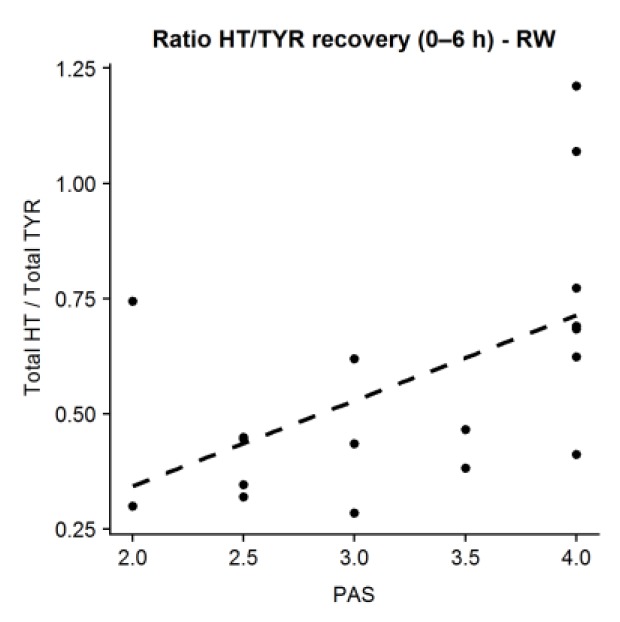
Correlation between polygenic activity score (PAS) and ratio HT:TYR of urinary recoveries (0–6 h) after red wine (RW) consumption.

There was a positive correlation between PAS and the ratio between total HT and total TYR at 0–6 h following RW consumption (Pearson’s r of 0.534, *p* = 0.022). Those subjects with greater PAS presented higher ratios (Figure 3). This linear association was not seen in the case of IPA, blonde, and free beers.

### 3.4. Effect of Urinary pH on TYR and HT Recovery

No influence of urinary pH on phenol excretion was observed in any of the urinary fractions after the consumption of the different beverages.

### 3.5. EtG Urinary Recovery

**Figure 4:**
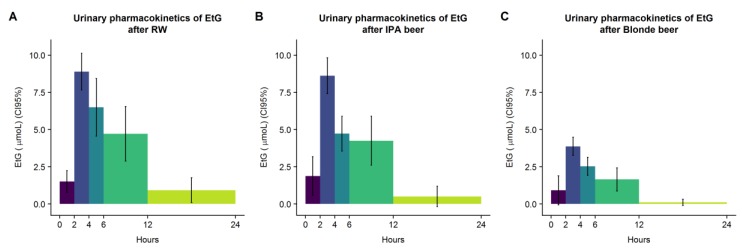
Urinary recovery of EtG after RW (**A**), IPA (**B**), and Blonde beers (**C**).

Baseline EtG levels were low (median and interquartile rank were 0 in all interventions) and did not differ significantly among interventions (*p* = 0.416). As shown in Table 6, 24 h EtG recovery was similar between RW and IPA beer, and significantly higher than blonde and free beers. Urinary kinetics of EtG followed a similar pattern in IPA and blonde beers compared to RW, in which the maximum peak was observed in the fraction 2–4 h and then declined (Figure 4). Finally, marginal sex differences in EtG recovery were observed following the intake of IPA beer (*p* = 0.058) and RW, in which women had higher levels than men (Table 6).

## 4. Discussion

The present study demonstrates that, in humans, the TYR present in beer is well absorbed and endogenously biotransformed into HT. Our results also suggest that alcoholic content and beer matrix composition, in addition to sex and genetics, are factors that affect the metabolic disposition of TYR and HT, and could help to understand the variability observed in the metabolism of these simple phenols.

We first analyzed whether the TYR in beer was as bioavailable as that found in RW. When the same dose of alcohol was administered (RW vs. IPA beer), the recovery of TYR (corrected by doses administered) was lower after beer intake (22.4 vs. 17.4, percental recovery). As previously reported, blonde beer was the treatment with highest recovery of the administered TYR (50.5%; CI95% 39.5%–61.5%), in the form of both TYR and HT, followed by RW, IPA, and free beers, which would suggest a matrix effect on TYR absorption. Beers are rich in other polyphenols and their content is known to vary widely among styles [19,20,21]. It is postulated that they could compete with TYR for their absorption, leading to a distinct TYR bioavailability. Remarkably, in our study, whilst free beer intake was associated with the lowest TYR recovery, the amount of TYR was similar to that present in the blonde beer although their alcohol content differed. Our findings confirm that the presence of alcohol is a key contributor to TYR bioavailability although not in a dose-dependent manner. The enhanced phenol absorption due to the presence of ethanol within the matrix has already been reported [6].

The present results confirm beer as a source of HT even though its presence was almost undetectable. We observed a positive correlation between the ingested dose of TYR and HT urinary recovery. These findings further support the hypothesis of TYR being an endogenous precursor of HT in vivo. Nonetheless, a higher HT recovery adjusted by the given dose was described following RW (11.3%) compared to beer intake (5.6% to 7.5%). It is known that alcohol consumption produces a shift in dopamine metabolism, resulting in endogenous HT formation as a byproduct of dopamine in a dose-response manner to the alcohol consumed [16]. This could explain part of the different HT recoveries following the intake of beverages with varying alcohol content. Nevertheless, the present differences in HT recovery suggest that TYR to HT conversion could be affected by other factors, as it was more efficient in RW than in IPA and blonde beers. A distinctive ingredient of beer is hops, especially rich in prenylated phenols. They have been reported to be potent and selective inhibitors of multiple members of the cytochrome P450 (CYP) family [22]. Interestingly, different beer extracts have been studied for their inhibitory effect on *CYP2D6*. Its activity was inhibited by all beer extracts with the greatest inhibition obtained with porter beer followed by ales whilst lager beers were the least inhibitors [23]. There is no study assessing hop constituent effect on *CYP2A6*. Based on these findings, hops present in beer could interfere in the TYR to HT conversion rate which would explain the lower production of HT observed following beer consumption and the lower impact of the genetic polymorphisms of these enzymes.

The urinary kinetics of TYR differs from HT in that the quantitative recovery of TYR is essentially concentrated in the first 0 to 4 h, while HT recovery is still relevant at 4 to 6 h. This is not surprising, since HT originates from the metabolic conversion of TYR into HT.

Our study confirms the extensive phase-II metabolism that TYR and HT undergo following their absorption. As has been described, once ingested, they go through an extensive first-pass hepatic metabolism, resulting in almost undetectable free forms in systemic biological fluids [24]. In our study, free forms of HT and TYR were excreted at very low concentrations compared to their metabolites. Previous authors have suggested that the low concentrations of the free forms observed in vivo cannot explain the biological activities associated with phenolic consumption in clinical and epidemiological studies [25]. Therefore, metabolites have been proposed as exhibiting relevant biological properties, either by having activity themselves or being deconjugated intracellularly giving rise to free active forms, as has been described for other dietary phenols [26,27]. In the present study we show an increase in TYR and HT phase II metabolite recovery following RW and beer treatments. The main metabolites recovered were TYR-4-sulfate, TYR-4-glucuronide, and HT-3-sulfate. They have been shown to possess in vitro biological activity equivalent to their parent compounds [28,29,30,31]. The fact that beer produced a rise in the same metabolites as RW suggests that it could trigger equivalent health effects.

The current work provides further evidence of the mediation of *CYP2A6* and *CYP2D6* on TYR to HT biotransformation [9,11]. PAS scores positively correlated with the amount of HT recovered (higher PAS are related to higher HT/TYR ratios in urine) after RW intake. Results were only observable in the case of RW, probably due to its higher TYR absorbed dose and the different composition in polyphenols compared to beer, as other factors than genetics have been described to affect the final activity of *CYP2A6* and *CYP2D6* enzymes (such as certain drugs and dietary and endogenous components) [10,14].

Sex differences in the metabolism of TYR and HT were observed in this study. Men were more efficient converting absorbed TYR to HT (higher HT/TYR ratio) after the administration of RW. This result is consistent with previous findings from the PREDIMED study, in which men presented higher values of HT adjusted by alcohol consumption [32]. Another clinical study administering olive leaf extracts reported greater concentrations of HT metabolites in men compared to women in response to the same treatment [33]. In our study, no significant sex differences were observed following IPA, blonde, and free beer treatments, suggesting that other factors could be affecting TYR to HT biotransformation capacity in women. It is well known that *CYP2A6* activity is upregulated by estrogen levels [10], indicating a higher activity in women compared to men; however, this was not observed in our study. Nonetheless, the influence of estrogen status and the fluctuation intrinsic to the menstrual cycle could affect *CYP2A6* activity distinctly among the experimental sessions which may explain the absence of correlation among treatment ratios in women. Caution should be taken when interpreting these results as women presented lower PAS values than men.

EtG is a widely used biomarker of alcohol consumption (and of compliance to alcohol abstention prior to experimental sessions) and as such was applied in this study [34]. Its recovery following our treatments matched their alcohol content. Interestingly, marginal differences were observed between genders in EtG recovery, particularly for the IPA beer. This observation would be in line with the existence of variations in alcohol metabolism between men and women [35].

Our findings enhance understanding of the antioxidant composition of beer and its potential health effects. Currently, beer is the most popular drink in the western world [36], whilst RW consumption is decreasing. Nevertheless, at similar given doses of TYR and equal doses of alcohol, RW exhibited a higher recovery on TYR and HT metabolites, suggesting a better bioavailability of TYR and a more efficient conversion to HT. Therefore, it is likely that the beneficial effects attributed to RW moderate consumption are superior to the observed following beer consumption. A large body of evidence exists about the health effects attributed to moderate RW consumption, whilst less is known about moderate beer consumption, and data are mostly derived from epidemiological studies [37]. The study of traditional beer and non-alcoholic beer can help to understand the role of ethanol and its interaction with beer polyphenols and their biological effects. Beer alcoholic content is lower than RW and, as shown in the present study, beer consumption triggered an increase in similar phenols. Non-alcoholic beer led to a mild increment in TYR and HT metabolites due to its lower bioavailability. Nevertheless, it could represent a source of these compounds in cases where alcohol consumption has to be limited.

Our study presents certain strengths and limitations. Firstly, the cross-over design enabled the observation of intra-individual changes, control for potential confounders, and between-subject variation. Secondly, we also evaluated the presence of sex differences in the metabolism of dietary phenolic compounds. This approach goes beyond traditional medical and nutritional studies that have long been performed in men and translated directly to women. Another strength of this study is that the administered doses of RW and beers are achievable in a normal diet and in agreement with moderate alcohol consumption. The current investigation was; however, limited by the low sample size which hampered the simultaneous grouping and comparison of volunteers according to their sex and PAS. This would have been relevant since PAS was not equally distributed between the genders. In addition, treatments were given at a single dose; it is possible that longer administration would have resulted in different results attributed to sex or PAS. Finally, further studies should be performed to assess the relevance of the clinical effects of TYR and endogenous HT formation associated with moderate beer consumption, at a postprandial level, and after chronic consumption.

## 5. Conclusions

In conclusion, our study demonstrates that beer is a source of TYR and an indirect source of HT. The HT content of beer is almost zero; nevertheless, there is an increase in HT urinary recovery after the consumption of beer following a dose-response relationship with the administered TYR. These results suggest a common mechanism between RW and beer consumption: TYR absorption and endogenous biotransformation to HT. Moreover, our results suggest that TYR bioavailability is increased by the presence of ethanol in the beer matrix. Finally, this mechanism could be modulated by sex, genetics, and the components of each beverage.

## Figures and Tables

**Table 1:** TYR, HT, and alcohol content of wine and beers administered in the study.

	Treatment			
	RW	IPA	Blonde	Free
TYR (mg/L)	25.30	9.70	4.20	3.30
HT (mg/L)	1.80	0.10	0.10	0.01
Alcohol (% vol/vol)	14.00	8.50	4.50	0.00
Dose (mL)	150	250	250	250
TYR administered (mg)	3.80	2.40	1.10	0.80
HT administered (mg)	0.30	0.03	0.01	0.00
Alcohol (g)	16.57	16.77	8.88	0.00

RW—red wine; IPA—IPA beer; Blonde—blonde beer; Free—non-alcoholic beer; TYR—tyrosol; HT—hydroxytyrosol.

**Table 2:** Activity scores assigned to the presence of *CYP2A6* and *CYP2D6* alleles.

Functionality	*CYP2A6*	*CYP2D6*	Activity Score
Non-functional	* 2, * 4	* 4, * 5	0
Reduced function	* 9, * 12	* 9, * 10, * 41	0.5
Functional	* 1	* 1, * 2, *35	1
Increased function	* 1 xN	* 1 xN, * 2 xN, * 35 xN	xN

xN indicates more than 1 copy. * indicates the allelic variants.

**Table 3:** Description of study participants.

	All Participants	Men	Women
*n* (%)	20 (100%)	10 (50%)	10 (50%)
Age (years)	24.3 (3.9)	23.9 (4.1)	24.7 (3.8)
BMI (kg/m^2^)	22.2 (2.0)	23.2 (2.0)	21.1 (1.4) **
Weight (kg)	66.6 (10.4)	73.0 (8.3)	60.3 (8.4) *
Baseline EtG (μmoL)	0.22 (1.5)	0.39 (2.07)	0.0 (0.0)

BMI—body mass index. Data is shown as mean (SD). Sex baseline comparisons were assessed by Student’s *t*-test for independent samples; * *p* < 0.050; ** *p* < 0.010 women vs. men.

**Table 4:** Total TYR and HT urinary recovery by fractions of 0–2, 2–4, 4–6, 6–12, and 12–24 h and the total recovery during the first 6 and 24 h post-administration of the different treatments. Results are expressed in μmoL.

			RW	IPA	Blonde	Free	ANOVA *		
			Mean (SD)	Mean (SD)	Mean (SD)	Mean (SD)	df	F	*p*
Total TYR	Accumulated	0–6 h	6.2 (2.9)	3.1 (2.4) ^w^	3.3 (1.8) ^w^	0.2 (0.5) ^w,i,b^	(3.55)	32.4	<0.001
		0–24 h	6.5 (2.9)	3.4 (2.5) ^w^	3.7 (1.8) ^w^	0.6 (0.8) ^w,i,b^	(3.55)	30.3	<0.001
	Fraction	0–2 h	4.1 (2.1)	2.2 (1.7) ^w^	2.6 (1.5) ^w^	0.2 (0.51) ^w,i,b^	(3.55)	23.3	<0.001
		2–4 h	1.9 (1.5)	0.7 (0.7) ^w^	0.5 (0.4) ^w^	0.03 (0.02) ^w,i^	(3.54)	21.8	<0.001
		4–6 h	0.3 (0.5)	0.1 (0.1)	0.1 (0.1)	0.04 (0.02)	(3.54)	3.2	0.031
		6–12 h	0.1 (0.1)	0.2 (0.1)	0.1 (0.1)	0.06 (0.05) ^i^	(3.55)	4.8	0.005
		12–24 h	0.3 (0.5)	0.2 (0.2)	0.2 (0.1)	0.3 (0. 6)	(3.55)	0.4	0.787
Total HT	Accumulated	0–6 h	3.1 (1.3)	1.0 (0.6) ^w^	0.6 (0.3) ^w^	0.4 (0.5) ^w^	(3.55)	55.3	<0.001
		0–24 h	4.8 (2.4)	2.8 (1.4) ^w^	1.5 (0.7) ^w^	2.3 (1.6) ^w^	(3.55)	15.2	<0.001
	Fraction	0–2 h	1.7 (1.2)	0.3 (0.2) ^w^	0.3 (0.2) ^w^	0.12 (0.4) ^w^	(3.55)	26.8	<0.001
		2–4 h	1.1 (0.7)	0.5 (0.6) ^w^	0.2 (0.1) ^w^	0.1 (0.1) ^w^	(3.54)	28.5	<0.001
		4–6 h	0.3 (0.3)	0.2 (0.2)	0.1 (0.11) ^w^	0.2 (0.2)	(3.54)	3.4	0.023
		6–12 h	0.6 (1.1)	0.7 (0.7)	0.3 (0.2)	0.8 (1.1)	(3.55)	1.4	0.245
		12–24 h	1.0 (0.9)	1.1 (1.2)	0.7 (0.5)	1.1 (0.86)	(3.55)	1.2	0.314

RW: red wine; IPA: IPA beer; Blonde: blonde beer; Free: non-alcoholic beer. SD: standard deviation; df: degrees of freedom; TYR: tyrosol; HT: hydroxytyrosol. Total TYR is the sum of free TYR, TYR-4-sulphate, and TYR-4-glucuronide. Total HT is the sum of free HT, HT-4-sulphate, HT-glucuronide (HT-3-glucuronide plus HT-4-glucuronide), HT-acetate-3-sulphate, HT-acetate, free Hval, and Hval-4-glucuronide. *ANOVA repeated measures. ^w, i, b^ = Tukey’s HSD post-hoc comparisons (W = *p* <0.05 compared to RW; I = *p* <0.05 compared to IPA; B = *p* <0.05 compared to blonde).

**Table 5:** Percentage of recovery for total TYR, total HT, and the sum of both compared to the dose of TYR administered (in μmoL) during the first 6 h post-administration of different treatments.

		%TYR (CI95%)	%HT (CI95%)	%TYR + HT (CI95%)
**0–6 h**	RW	22.4 (21.4–23.4)	11.3 (9.2–13.4)	33.8 (27.7–39.8)
	IPA	17.4 (16.1–18.8)	5.6 (3.9–7.2)	23.0 (16.3–29.7)
	Blonde	43.0 (40.5–45.5)	7.5 (5.9–9.1)	50.5 (39.5–61.5)
	Free	3.5 (2.6–4.4)	6.1 (2.7–9.6)	9.6 (2.6–16.7)

RW—red wine; IPA—IPA beer; Blonde—blonde beer; Free—non-alcoholic beer; CI95%—95% confidence interval; TYR—total tyrosol representing the sum of free TYR, TYR-4-sulphate, and TYR-4-glucuronide; HT—total hydroxytyrosol representing the sum of free HT, HT-4-sulphate, HT-glucuronide (HT-3-glucuronide plus HT-4-glucuronide), HT-acetate-3-sulphate, HT-acetate, free Hval, and Hval-4-glucuronide.

**Table 6:** Ethyl Glucuronide (EtG) urinary recovery (0–24 h). Results are expressed in μmoL.

	RW	IPA	Blonde	Free	ANOVA *		
	Mean (SD)	Mean (SD)	Mean (SD)	Mean (SD)	*df*	*F*	*P*
All sample	22.1 (7.9)	19.7 (7.0)	9.1 (3.7) ^w,i^	0.2 (1.1) ^w,i,b^	(3.55)	87.7	<0.001
Men	21.2 (8.7)	16.8 (7.0)	9.5 (4.0) ^w,i^	0.5 (1.5) ^w,i,b^	(3.25)	36.2	<0.001
Women	23.0 (7.4)	22.7 (6.1)	8.7 (3.5) ^w,i^	0.0 (0.0) ^w,i,b^	(3.27)	59.3	<0.001

RW—red wine; IPA—IPA beer; Blonde—blonde beer; Free—non-alcoholic beer. SD—standard deviation; df—degrees of freedom; TYR—tyrosol; HT—hydroxytyrosol. * ANOVA repeated measures. ^w, i, b^ = Tukey’s honestly significant difference (HSD) post-hoc comparisons (W = *p* < 0.05 compared to RW; I = *p* < 0.05 compared to IPA; B = *p* < 0.05 compared to Blonde).

## Supplementary Materials

The following are available online at https://www.mdpi.com/2072-6643/11/9/2241/s1, Detailed description 1: Low-phenol content diet during the study, Table S1: TYR and HT metabolite urinary recovery during the first 6 and 24 h post-administration of different treatments, Table S2: TYR and HT metabolite urinary recovery by fractions of 0–2, 2–4, 4–6, 6–12, and 12–24 h post-administration of different treatments.
