# Supplementary material for: Generation of the Antioxidant Hydroxytyrosol from Tyrosol Present in Beer and Red Wine in a Randomized Clinical Trial
Source: Nutrients. 2019 Sep 18;11(9):2241. doi: 10.3390/nu11092241 (PMC6769679; doi:10.3390/nu11092241)
Supplement: Supplementary file 1 [file nutrients-11-02241-s001.pdf]

nutrients MDPI

Detailed description 1: Low-phenol content diet during the study

Study participants were asked to follow a controlled diet with a low antioxidant content, and to abstain from the following foods and beverages throughout the study: alcoholic drinks (except within the framework of treatment allocations), vinegar and foods derived from olives or containing olives (e.g., olives, olive paté, olive oil, canned products in olive oil, pre-cooked products with olive oil). The consumption of coffee and energy drinks was limited to one serving/day. The use of cosmetics that contained alcohol (e.g., deodorant, colognes) was forbidden on the day of the session until the end of urine collection. Other prohibitions included the use of mouthwashes with alcohol, cosmetics with olive oil, performing strenuous physical exercise, and taking any type of drug.

Table 1. TYR and HT metabolite urinary recovery during the first 6 and 24 h post-administration of different treatments. Results are expressed in μmoL.

		RW	IPA	Blonde	Free	ANOVA *		
		**Mean(SD)**	**Mean(SD)**	**Mean(SD)**	**Mean(SD)**	***df***	***F***	***p-value***
0–6 h	Free TYR	0.40 (0.37)	0.24 (0.27)	0.26 (0.26)	0.01 (0.02) ^{w,i,b}	(3.55)	10.4	<0.001
	TYR-s	2.27 (1.57)	0.92 (0.8) ^{w}	1.03 (0.99) ^{w}	0.08 (0.24) ^{w,i,b}	(3.55)	18.7	<0.001
	TYR-g	3.49 (1.25)	1.89 (1.64) ^{w}	2.00 (0.83) ^{w}	0.13 (0.27) ^{w,i,b}	(3.55)	37.3	<0.001
	Free HT	0.17 (0.19)	0.07 (0.08) ^{w}	0.05 (0.07) ^{w}	0.04 (0.06) ^{w}	(3.55)	8.7	<0.001
	HT-s	2.51 (1.37)	0.57 (0.56) ^{w}	0.37 (0.25) ^{w}	0.16 (0.36) ^{w}	(3.55)	45.9	<0.001
	HT-g	0.12 (0.05)	0.06 (0.05) ^{w}	0.04 (0.04) ^{w}	0.04 (0.04) ^{w,i}	(3.55)	43.1	<0.001
	HT-ac	0.00 (0.00)	0.00 (0.00)	0.00 (0.01)	0.00 (0.01)	(3.55)	0.6	0.636
	HT-ac-s	0.02 (0.04)	0.03 (0.10)	0.00 (0.01)	0.02 (0.04)	(3.55)	1.3	0.296
	HVAL	0.15 (0.14)	0.16 (0.34)	0.06 (0.09)	0.10 (0.19)	(3.55)	1.0	0.397
	HVAL-g	0.15 (0.10)	0.09 (0.07) ^{w}	0.04 (0.04) ^{w,i}	0.01 (0.03) ^{w,i}	(3.55)	26.7	<0.001
0–24 h	Free TYR	0.41 (0.38)	0.25 (0.28)	0.26 (0.26)	0.02 (0.09) ^{w,i,b}	(3.55)	9.7	<0.001
	TYR-s	2.47 (1.65)	1.06 (0.79) ^{w}	1.16 (0.97) ^{w}	0.18 (0.29) ^{w,b}	(3.55)	19.2	<0.001
	TYR-g	3.65 (1.26)	2.12 (1.78) ^{w}	2.23 (0.84) ^{w}	0.41 (0.5) ^{w,i,b}	(3.55)	30.0	<0.001
	Free HT	0.30 (0.32)	0.14 (0.17) ^{w}	0.10 (0.10) ^{w}	0.26 (0.29) ^{b}	(3.55)	5.0	0.004
	HT-s	3.00 (1.62)	1.50 (1.17) ^{w}	0.89 (0.68) ^{w}	0.80 (0.80) ^{w}	(3.55)	19.8	<0.001
	HT-g	0.28 (0.38)	0.19 (0.19)	0.12 (0.12)	0.19 (0.25)	(3.55)	2.0	0.132
	HT-ac	0.02 (0.06)	0.02 (0.05)	0.02 (0.05)	0.04 (0.08)	(3.55)	0.5	0.651
	HT-ac-s	0.07 (0.2)	0.08 (0.20)	0.07 (0.13)	0.08 (0.14)	(3.55)	0.1	0.984
	HVAL	0.82 (1.44)	0.59 (0.67)	0.25 (0.3)	0.74 (1.17)	(3.55)	1.4	0.25
	HVAL-g	0.29 (0.33)	0.27 (0.32)	0.11 (0.12)	0.18 (0.30)	(3.55)	2.8	0.047

RW—red wine; IPA—IPA beer; Blonde—blonde beer; Free—non-alcoholic beer. TYR-s—TYR-4-sulphate; TYR-g—TYR-4-glucuronide; HT-s—HT-4-sulphate; HT-g—HT-glucuronide (HT-3-glucuronide plus HT-4-glucuronide); HT-ac-s—HT-acetate-3-sulphate; HT-ac—HT-acetate, HVAL—free Hval; HVAL-g—Hval-4-glucuronide. * ANOVA repeated measures. ^{w,i,b}= Tukey's HSD post-hoc comparisons(W = p < 0.05 compared to RW; I = p < 0.05 compared to IPA; B = p < 0.05 compared to blonde).

Table 2. TYR and HT metabolite urinary recovery by fractions of 0–2, 2–4, 4–6, 6–12, and 12–24 h post-administration of different treatments. Results are expressed in μmoL.

	Fraction	RW	IPA	Blonde	Free
		**Mean(SD)**	**Mean(SD)**	**Mean(SD)**	**Mean(SD)**
Free TYR	0–2 h	0.35 (0.31)	0.21 (0.25)	0.24 (0.22)	0.01 (0.02)
	2–4h	0.05 (0.09)	0.02 (0.04)	0.02 (0.03)	0.00 (0.01)
	4–6h	0.00 (0.01)	0.00 (0.01)	0.01 (0.03)	0.00 (0.00)
	6–12h	0.01 (0.05)	0.01 (0.03)	0.00 (0.00)	0.00 (0.00)
	12-24h	0.00 (0.01)	0.00 (0.00)	0.00 (0.00)	0.02 (0.08)
TYR-s	0–2 h	1.47 (1.17)	0.66 (0.64)	0.86 (0.89)	0.06 (0.23)
	2–4h	0.76 (0.71)	0.23 (0.22)	0.14 (0.14)	0.01 (0.01)
	4–6h	0.08 (0.15)	0.04 (0.05)	0.03 (0.03)	0.01 (0.01)
	6–12h	0.03 (0.04)	0.06 (0.06)	0.06 (0.09)	0.02 (0.03)
	12–24h	0.17 (0.51)	0.07 (0.07)	0.06 (0.08)	0.08 (0.10)
TYR-g	0–2 h	2.27 (0.98)	1.35 (1.11)	1.53 (0.62)	0.08 (0.26)
	2–4h	1.09 (0.82)	0.45 (0.51)	0.37 (0.31)	0.02 (0.02)
	4–6h	0.18 (0.31)	0.09 (0.08)	0.10 (0.08)	0.03 (0.02)
	6–12h	0.06 (0.04)	0.08 (0.06)	0.07 (0.05)	0.04 (0.05)
	12–24h	0.10 (0.10)	0.16 (0.25)	0.16 (0.11)	0.24 (0.44)
Free HT	0–2 h	0.09 (0.11)	0.04 (0.06)	0.04 (0.06)	0.01 (0.03)
	2–4h	0.05 (0.07)	0.02 (0.02)	0.01 (0.02)	0.01 (0.02)
	4–6h	0.03 (0.04)	0.01 (0.01)	0.01 (0.01)	0.02 (0.04)
	6–12h	0.10 (0.22)	0.03 (0.05)	0.02 (0.04)	0.11 (0.23)
	12–24h	0.03 (0.06)	0.05 (0.08)	0.03 (0.07)	0.11 (0.18)
HT-s	0–2 h	1.47 (1.19)	0.16 (0.14)	0.2 (0.2)	0.08 (0.34)
	2–4h	0.89 (0.71)	0.32 (0.57)	0.1 (0.08)	0.01 (0.01)
	4–6h	0.2 (0.29)	0.1 (0.09)	0.07 (0.09)	0.07 (0.1)
	6–12h	0.2 (0.32)	0.35 (0.39)	0.14 (0.18)	0.2 (0.28)
	12-24h	0.29 (0.42)	0.57 (0.99)	0.38 (0.44)	0.44 (0.63)
HT-g	0–2 h	0.05 (0.03)	0.02 (0.02)	0.01 (0.02)	0.01 (0.02)
	2–4h	0.05 (0.03)	0.02 (0.01)	0.01 (0.01)	0.01 (0.01)
	4–6h	0.03 (0.02)	0.02 (0.02)	0.02 (0.02)	0.02 (0.03)
	6–12h	0.02 (0.03)	0.05 (0.05)	0.03 (0.07)	0.05 (0.09)
	12–24h	0.13 (0.38)	0.08 (0.11)	0.04 (0.05)	0.1 (0.22)
HT-ac	0–2 h	0.00 (0.00)	0.00 (0.00)	0.00 (0.00)	0.00 (0.00)
	2–4h	0.00 (0.00)	0.00 (0.00)	0.00 (0.01)	0.00 (0.00)
	4–6h	0.00 (0.00)	0.00 (0.00)	0.00 (0.00)	0.00 (0.01)
	6–12h	0.00 (0.00)	0.01 (0.02)	0.00 (0.00)	0.01 (0.02)
	12–24h	0.02 (0.06)	0.01 (0.04)	0.02 (0.05)	0.03 (0.07)
HT-ac-s	0–2 h	0.00 (0.00)	0.00 (0.02)	0.00 (0.00)	0.00 (0.00)
	2–4h	0.00 (0.00)	0.02 (0.08)	0.00 (0.00)	0.00 (0.00)
	4–6h	0.02 (0.03)	0.01 (0.03)	0.00 (0.01)	0.02 (0.04)
	6–12h	0.00 (0.02)	0.01 (0.02)	0.02 (0.08)	0.02 (0.07)
	12–24h	0.04 (0.18)	0.04 (0.17)	0.04 (0.11)	0.03 (0.09)
HVALC	0–2 h	0.05 (0.07)	0.06 (0.14)	0.02 (0.05)	0.02 (0.06)
	2–4h	0.05 (0.06)	0.04 (0.11)	0.02 (0.04)	0.02 (0.05)
	4–6h	0.05 (0.06)	0.06 (0.12)	0.01 (0.03)	0.06 (0.13)
	6–12h	0.26 (0.86)	0.20 (0.39)	0.07 (0.11)	0.38 (0.78)
	12–24h	0.41 (0.77)	0.23 (0.33)	0.12 (0.24)	0.25 (0.52)
HVALC-g	0–2 h	0.06 (0.06)	0.03 (0.04)	0.01 (0.02)	0.01 (0.02)
	2–4h	0.07 (0.05)	0.04 (0.03)	0.02 (0.02)	0.00 (0.01)
	4–6h	0.03 (0.03)	0.02 (0.03)	0.01 (0.02)	0.00 (0.01)
	6–12h	0.05 (0.11)	0.08 (0.19)	0.02 (0.04)	0.04 (0.11)
	12–24h	0.08 (0.19)	0.10 (0.15)	0.04 (0.07)	0.12 (0.22)

RW—red wine; IPA—IPA beer; Blonde—blonde beer; Free—non-alcoholic beer. TYR-s—TYR-4-sulphate; TYR-g—TYR-4-glucuronide; HT-s—HT-4-sulphate; HT-g—HT-glucuronide (HT-3-glucuronide plus HT-4-glucuronide); HT-ac-s—HT-acetate-3-sulphate; HT-ac—HT-acetate, HVAL—free Hval; HVAL-g—Hval-4-glucuronide.

© 2019 by the authors. Licensee MDPI, Basel, Switzerland. This article is an open access article distributed under the terms and conditions of the Creative Commons Attribution (CC BY) license (http://creativecommons.org/licenses/by/4.0/).
